# 24-hour movement behaviour composition associated with desired social-emotional functioning in the early years

**DOI:** 10.1371/journal.pone.0361046

**Published:** 2026-10-21

**Authors:** Samah Zahran, Dylan P. Cliff, Devan Antczak, Eivind Aadland, Emad A. Anam, Katrine N. Aadland, Jade Burley, Catherine E. Draper, Dorothea Dumuid, Diego Augusto Santos Silva, Esther M. F. van Sluijs, Hayley Christian, Mark S. Tremblay, Timothy S. Olds, Rute Santos, Zhiguang Zhang, Ian Janssen

**Affiliations:** 1 School of Kinesiology and Health Studies, Queen’s University, Kingston, Ontario, Canada; 2 School of Education, Early Start, Faculty of the Arts, Social Sciences & Humanities, University of Wollongong, Wollongong, Australia; 3 Department of Sport, Food and Natural Sciences, Faculty of Education, Arts and Sports, Western Norway University of Applied Sciences, Sogndal, Norway; 4 Department of Orthopedic Surgery, King Abdulaziz University, Jeddah, Saudi Arabia; 5 SAMRC Developmental Pathways for Health Research Unit, Department of Paediatrics, Faculty of Health Sciences, University of the Witwatersrand, Johannesburg, South Africa; 6 Alliance for Research in Exercise, Nutrition and Activity (ARENA), Allied Health & Human Performance, University of South Australia, Adelaide, Australia; 7 Department of Physical Education. Federal University of Santa Catarina, Sports Center, Florianopolis, Santa Catarina, Brazil; 8 MRC Epidemiology Unit, School of Clinical Medicine, University of Cambridge, Cambridge, United Kingdom; 9 School of Population and Global Health and Telethon Kids Institute, The University of Western Australia, Perth, Western Australia, Australia; 10 Healthy Active Living and Obesity Research Group, Children’s Hospital of Eastern Ontario Research Institute, Ottawa, Ontario, Canada; 11 Department of Pediatrics, University of Ottawa, Ottawa, Ontario, Canada; 12 Faculty of Kinesiology, Sport, and Recreation, University of Alberta, Edmonton, Canada; 13 Institute of Education and Research Centre on Child Studies, University of Minho, Braga, Portugal; 14 Department of Public Health Sciences, Queen’s University, Kingston, Ontario, Canada; UBC: The University of British Columbia, CANADA

## Abstract

**Introduction:**

Movement behaviours (sleep, sedentary behaviour, and physical activity) influence numerous health outcomes in children, including their social-emotional development. The optimal 24-hour movement behaviour composition for positive social-emotional development is unknown. The purpose of this study was to determine the movement behaviour composition across the 24-hour day associated with desired social-emotional functioning in the early years.

**Methods:**

This cross-sectional analysis included 560 children aged 1.2–2.9 years and 1,500 children aged 3.0–4.9 years from the Sleep and Activity Database (SADEY) for the Early Years study. Sedentary behaviour (SED), light physical activity (LPA), and moderate-to-vigorous physical activity (MVPA) were measured using ActiGraph accelerometers. Sleep duration was assessed using parent reports. Children social-emotional functioning was measured using the five domains of the Strengths and Difficulties Questionnaire (SDQ), a widely used screening instrument of children’s behaviours, emotions, and relationships across five domains (emotional problems, conduct problems, hyperactivity, peer problems, and prosocial behaviour). Compositional data analyses were used to estimate the 24-hour movement behaviour compositions associated with optimal scores for each of the SDQ domain z-scores and all five domains combined (the Goldilocks day).

**Results:**

The movement behaviour composition was significantly associated with all five SDQ domains (all p < .01). For 1–2-year-olds, the Goldilocks day consisted of 12 h 12 min of sleep, 4 h 36 min of SED, 5 h 48 min of LPA, and 1h 24 min of MVPA. For 3–4-year-olds, it consisted of 12 h 08 min of sleep, 4 h 27 min of SED, 5 h 49 min of LPA, and 1 h 36 min of MVPA.

**Conclusion:**

The duration of total physical activity and MVPA associated with more favourable social-emotional functioning exceeded the minimum levels specified in the current 24-hour Movement Guidelines. These findings should not be interpreted as behavioural recommendations but rather as movement behaviour patterns associated with more favourable outcomes within this study population.

## Introduction

The amount of time young children spend sleeping, being sedentary, and engaging in different intensities of physical activity (PA) is related to various health outcomes, including their social-emotional development [1–5]. The World Health Organization recommends that children aged 1–2 years sleep for 11–14 hours per day, limit sedentary screen time to 1 hour per day or less if aged 2 years or avoid screen time if younger than 2 years, and accumulate at least 3 hours per day of PA [6]. It is recommended that children aged 3–4 years should sleep for 10–13 hours per day, limit sedentary screen time to 1 hour per day or less, and accumulate at least 3 hours per day of PA, including at least 1 hour of moderate-to-vigorous-intensity PA (MVPA) [6].

The recommendations mentioned above were developed using a comprehensive approach that considered all movement behaviours [6,7]. However, these recommendations were mostly derived from studies that used statistical approaches that treated movement behaviours as independent of each other despite their inherent interdependence within the 24-hour day, which might misrepresent associations [4]. Moreover, although these recommendations offer guidance on a healthy range of movement behaviours, they do not provide information about the healthiest movement behaviour time-use composition across the full 24-hour day, which may vary for different health outcomes. Understanding the healthiest time compositions could help tailor movement behaviour recommendations to individual health goals.

Compositional data analysis (CoDA) provides an analytical framework that allows movement behaviour researchers to properly account for the interrelated nature of movement behaviours within a 24-hour day. Because time is finite, increasing time spent in one movement behaviour necessarily requires decreasing time spent in one or more other behaviours. CoDA accounts for this co-dependence by examining movement behaviours relative to one another, rather than treating the time spent in each behaviour as an independent exposure [4]. A small but growing number of studies have applied CoDA to examine movement behaviours simultaneously in relation to health outcomes in early childhood. For example, Li et al. [8] found significant associations between the overall movement behaviour composition, as well as specific components of that composition, and social-emotional outcomes – including prosocial behaviors and externalizing problems – among 205 Chinese children aged 3–6 years. In contrast, other CoDA studies of early years children, including those by Phillips et al. [9] and Kuzik et al. [10], reported largely null associations for the overall movement behaviour composition, with associations limited to specific movement behaviour components or time-reallocation estimates with select social-emotional domains. Given the inconsistencies across studies, further research is needed to provide a better understanding of the relationships between movement behaviours and social-emotional development in early childhood.

In addition, CoDA can be used to determine the movement behaviour time-use composition associated with the healthiest outcomes, the so-called “*Goldilocks day*” which is a mixture of movement behaviours that is “just right” [11,12]. To our knowledge, only one study of early years children has used the CoDA Goldilocks day approach to examine associations between movement behaviours and measures of cognition and social-emotional health [13]. In that cross-sectional study of 388 children aged 2.7–5.8 years, the movement behaviour composition was associated with receptive vocabulary but not five other cognitive and social-emotional health measures. The *optimal* daily time-use composition for vocabulary scores consisted of 12.1 hours of sleep, 4.7 hours of SED, 5.6 hours of light physical activity (LPA), and 1.7 hours of MVPA [13]. However, the study was limited by a relatively small sample size and the inclusion of a wide developmental age range into the same *Goldilocks day* analysis. Additional research using CoDA *Goldilocks day* analyses, with larger and more diverse sample sizes of early years children, could provide further evidence to inform movement behaviour guidelines.

The objective of this study was to use CoDA to explore the *Goldilocks day* movement behaviour time-use composition for social-emotional functioning in the early years (ages 1–2 years and 3–4 years).

## Methods

### Study design and participants

Data were from the Sleep and Activity Database for the Early Years (SADEY) [14]. SADEY is an ongoing international collaborative collection of pooled accelerometer-measured movement behaviour data in 8,409 children aged 7 years or younger, from 14 studies, collected in 7 countries between 2009 and 2022 [14]. Ethics approval was obtained for the SADEY project from the University of Wollongong Health and Medical Human Research Ethics Committee (2021/249). Each contributing study was also approved by its respective Human Research Ethics Committee as follows: ACTNOW – Institutional Ethics Committee and the Norwegian Centre for Research Data - #248220; GET-UP – University of Wollongong Human Research Ethics Committee (HE15/236); PATH ABC – University of Wollongong Health and Medical Human Research Ethics Committee - #HE14/310); PLAYCE – The University of Western Australia Human Ethics Research Committee (RA/4/1/7417). Data were accessed between January 2022 and the present for research purposes. For each study, written informed consent was obtained from a parent or legal guardian of each child participant. Assent was not required due to the participants’ young age (under 5 years). All data were anonymized prior to analysis.

In each study, children wore an Actigraph accelerometer on their waist for at least one day. Accelerometer data from all contributing studies were re-processed in a harmonized manner. Additionally, measures of several health and developmental outcomes and sociodemographic covariates were harmonized and pooled, although the availability of these measures differs between the studies.

For the present study, cross-sectional data from four contributing SADEY studies were included: the Preschool Activity Technology, Health, Adiposity, Behaviour and Cognition (PATH-ABC) study conducted in Wollongong, Australia [15]; the Active Learning Norwegian Preschool(er)s (ACTNOW) study conducted in Sogndal, Norway [16]; the Get-Up study conducted in Wollongong, Australia [17]; and the Play Spaces and Environments for Children’s Physical Activity (PLAYCE) study conducted in Western Australia [18]. Because we were interested in the early years as defined in the World Health Organization guidelines on physical activity, sedentary behaviour, and sleep [6)] our sample size was limited to SADEY participants < 5 years of age. Furthermore, because of the developmental differences across the early years, participants were stratified into 1–2-year-old and 3–4-year-old categories, which correspond to the age categories used in the World Health Organization guidelines. A posthoc power analysis conducted in G*power using an alpha of 0.05 and an effect size of 0.15 indicated that our study achieved 100% power in each age group.

### Movement behaviours

Sleep duration over the full 24-hour day, including naps, was assessed by parent report. The PATH-ABC, ACTNOW, and PLAYCE studies included a single questionnaire item that assessed sleep duration. The Get-Up study included items that assessed bedtime and wake-up times and responses were used to estimate sleep duration. A recent study showed that parent- reported sleep duration of children aged 3–6 years is highly correlated to device-based measures (r = 0.8); however, the average parental reported values were approximately 4% (37 min/day) higher [19].

Average daily time spent in SED, LPA, and MVPA were measured using ActiGraph GT3X+ accelerometers (ActiGraph Corporation, Pensacola FL) worn on the right hip using an elastic belt. Accelerometer data were processed using the PhysicalActivity package [20] in R version 4.2.2 [21]. As per the SADEY accelerometer data processing protocol [14], raw accelerometer data were collapsed into 15-second epochs. Additionally, in line with commonly accepted decisions for this age group [14], non-wear time was identified as ≥20 minutes of consecutive zero counts [22]. At least 6 hours of wear time during waking hours were required for a day to be considered valid [23]. Days were defined as midnight to midnight. In studies that used a 24-hour wear protocol, time spent sleeping was removed from the accelerometer data before assessing waking time movement behaviours [14]. To be included in the analysis, participants were required to have ≥ 3 valid days, with no restrictions on specific days of the week [23]. In our dataset, the number of valid days for participants included in the analysis ranged from 3 to 7 days, with an average of 5 days. In young children, ≥ 6 hours of wear time on any 3 days provides reliable estimates of movement behaviours (intraclass correlation coefficient = 0.7) [24]. A cut-point of ≤ 25 counts per 15-seconds was used to distinguish SED from LPA [25] and a cut-point of ≥ 420 counts per 15 seconds was used to distinguish MVPA from LPA [26]. These cut-points are more accurate at classifying movement intensity in the early years than other cut-points commonly used in the literature [27].

The ActiGraph demonstrates acceptable reliability in early years children when estimating the consistency of accelerometer estimates of PA and SED across days from a single measurement period [23,28]. When comparing measurement periods over multiple seasons, the ActiGraph shows moderate reliability [29].

The average sleep duration (including nighttime sleep and naps) was 10 h 51 min ± 1 h 03 min for 1–2-year-olds and 10 h 40 min ± 0 h 59 min for 3–4-year-olds. The average ± SD waking wear times (sum of SED, LPA and MVPA) were 9 h 01 min ± 2 h 02 min and 9 h 55 min ± 2 h 06 min for the two age groups. Non-wear time was imputed by linearly adjusting the time spent in the waking behaviours to sum to 24 hours minus sleep duration, so that each participant’s averaged daily total movement behaviour time added up to exactly 24 hours [30]. This imputation assumed that non-wear time occurred during waking hours and was redistributed proportionally across SED, LPA, and MVPA based on their distribution during wear time. The average and range (minimum to maximum) imputed time for 1–2-year-olds was 1 h 47 min (1 h 1 min to 6 h 15 min) for SED, 1 h 47 min (1 h 10 min to 6 h 55 min) for LPA, and 24 min (11 min to 2 h 22 min) for MVPA. For 3–4-year-olds, the average imputed time was 1 h 29 min (1 h 23 min to 6 h 36 min) for SED, 1 h 34 min (1 h 16 min to 2 h 25 min) for LPA, and 22 min (15 min to 3 h 36 min) for MVPA.

### Social-emotional functioning

In all contributing SADEY studies, children’s social-emotional functioning was assessed using the Strengths and Difficulties Questionnaire (SDQ), a well-established screening instrument originally developed to identify emotional and behavioural difficulties in children [31,32]. Although the SDQ is not intended to provide a comprehensive assessment of socio-emotional functioning across developmental stages, it remains one of the most widely used and validated screening instruments for identifying children’s behaviours, emotions, and relationships in large studies of young children. In most SADEY studies, the SDQ was completed by teachers, except the PLAYCE study, where parents completed the questionnaire. The SDQ is a 25-item caregiver-completed questionnaire that evaluates five domains: hyperactivity, emotional symptoms, conduct problems, peer relationship problems, and prosocial behaviour [33]. Each domain has five questions with ‘not true’, ‘somewhat true’, and ‘certainly true’ response options. As per the SDQ protocol, scores of 0, 1, or 2 were assigned to the response options, with some items being reverse-coded (33). Scores from all five items for each domain were summed to create summary scores with a range of 0–10 [33]. Higher summary scores indicate poorer social-emotional health and development, except for prosocial behaviour where higher scores are favourable. Refer to S1 Table in S1 File for the SDQ items and scoring protocol.

Given that SDQ scores differ between boys and girls and vary with age due to normal growth and maturation, sex- and age-adjusted values were generated for analysis. To achieve this, each of the five SDQ scores was regressed onto a cubic polynomial of age in months (age, age^2^, and age^3^) separately for boys and girls. The standardized residuals from these regressions were retained, representing the sex- and age-adjusted values for subsequent analyses [34].

The SDQ questionnaire items have good reliability and structural validity [35,36]. The internal consistency determined using coefficient omega for the five SDQ factors was > 0.80 in Norwegian children aged 3–6 years (emotional symptoms ω = 0.85, conduct problems ω = 0.80, hyperactivity ω = 0.90, peer problems ω = 0.86, and prosocial behaviour ω = 0.90) [35,36]. Evidence also supports the psychometric properties of the preschool SDQ in younger children. In a large cohort of 2-year-olds, a modified five-factor structure was supported and internal consistency was satisfactory for four of the five subscales (α = 0.71–0.84), although internal consistency was lower for peer problems (α = 0.54) [37]. Research in children aged 1–2 years has also demonstrated acceptable overall psychometric properties, including internal consistency, test–retest reliability, and structural validity, although performance was generally stronger among older toddlers and for externalizing behaviours [32].

### Covariates

Parental marital status (single or dual parent household), highest parental education (≤ high school or > high school), and study (PATH-ABC, ACTNOW, Get-Up, or PLAYCE) were controlled for as confounding variables in regression analyses. We did not control for sex and age because the SDQ z-scores were standardized for these characteristics. The selection of confounding variables was limited to those collected across all contributing studies and variables that could be meaningfully harmonized. The harmonization of the parental status and education variables is described in S2 Table in S1 File.

### Statistical analysis

Statistical analyses were conducted in R 4.2.2 [21]. Conventional descriptive statistics were explored for the variables of interest based on SDQ raw scores. Geometric means for time spent in each movement behaviour were calculated.

We followed the Dumuid et al. [12] multi-step CoDA approach to estimate the Goldilocks day movement behaviour time-use composition for social-emotional development. In the initial step, multiple linear regression models were used to examine the relationships between the movement behaviour composition and the five domains of sex- and age-adjusted SDQ z-scores. This was done after transforming the time spent in sleep, SED, LPA, and MVPA using an isometric log-ratio coordinate (ilr) system prior to the analysis [38]. For illustration, as the original composition consists of four parts (sleep, SED, LPA, and MVPA), three ilr transformed variables [𝑧𝑖1, 𝑧𝑖2, 𝑧𝑖3] for each movement behaviour were created based on sequential partition. As an example, for MVPA the three ilr transformed variables can be written as:


$$\begin{array}{c} zi1=\sqrt{\frac{\text{3}}{\text{4}\;}}In\frac{MVPA}{\sqrt[{\text{3}}]{LPA\times SED\times sleep}},zi2=\sqrt{\frac{\text{2}}{\text{3}\;}}In\frac{LPA}{\sqrt[{\text{2}}]{SED\times sleep}},and\text{\textasciitilde{}}zi\text{3}=\sqrt{\frac{\text{1}}{\text{2}\;}}In\frac{SED}{\sqrt[{\text{1}}]{sleep}} \end{array}$$

To obtain behaviour-specific CoDA coefficients, the sequential binary partition was rotated four times so that each movement behaviour (sleep, SED, LPA, and MVPA) was placed in the numerator of the first ilr coordinate in turn. Separate regression models were then fitted for each partition, and the coefficient corresponding to the first ilr coordinate was reported as the behaviour-specific association of that movement behaviour relative to the remaining three movement behaviours.

All regression models were adjusted for the confounding variables. Quadratic terms for the ilr coordinates were tested using orthogonal polynomial regression to explore non-linear relationships. Quadratic terms were retained if they significantly improved model fit. R^2^ was used to determine the goodness-of-fit of the models.

In the second step, a grid of data points in 5-minute increments was created using the ring-fencing approach proposed by Nikfarjam et al. [39]. This grid represented all possible combinations of the four movement behaviours across a full day (1440 minutes). The ring-fencing approach involved calculating the mean vector and covariance matrix of the ilr transformed data. These parameters were then used to compute the Mahalanobis distance for each grid point in the ilr-transformed space, indicating its distance from the centre of the data distribution. Thresholds for the fence were tested at intervals between the 70th and 90th percentiles in 5-percentile increments. Visual inspection of the resulting prediction grids indicated that the 80th percentile captured the most representative time-use compositions of the sample, aligning with the areas of highest data density in the ilr-transformed space. Lower thresholds excluded many plausible observed movement behaviour compositions, whereas higher thresholds included increasingly peripheral combinations. Therefore, the 80th percentile was chosen for the final analyses. After applying the fencing, the resulting grid data contained 17,577 possible time-use compositions in 1–2-year-olds, and 16,535 in 3–4-year-olds. The range (minimum to maximum) of this grid for 1–2-year-olds was 8 h 40 min to 13 h 10 min for sleep, 4 h 0 min to 7 h 55 min for SED, 4 h 30 min to 7 h 30 min for LPA, and 50 min to 2 h 40 min for MVPA. For 3–4-year-olds, the range was 8 h 40 min to 12 h 50 min for sleep, 3 h 55 min to 7 h 40 min for SED, 4 h 45 min to 7 h 35 min for LPA, and 55 min to 2 h 55 min for MVPA.

In the third step, the regression models created in step 1 were used to predict the SDQ z-score for each domain for each predictive time-use composition generated in step 2 (retained within the ring fence). For each SDQ domain, the *best* time-use zone was operationally defined as the movement behaviour compositions corresponding to the most favourable 10% of predicted scores (i.e., the lowest predicted scores for emotional symptoms, conduct problems, hyperactivity, and peer problems, and the highest predicted scores for prosocial behaviour). The *optimal* time-use composition for each SDQ domain was then defined as the statistical centre (compositional mean) of these movement behaviour compositions.

Fourth, the *best* time-use zone was defined for each of the five SDQ z-scores as the predictive time-use compositions associated with the most favourable 10% of predicted SDQ scores. Then the *optimal* time-use composition for each of the five SDQ z-scores was defined as the centre (compositional mean) of the *best* time-use zone. In the next step, the *best of the best* time-use zone was defined as the overlapping areas of the *best* time-use zones for the individual SDQ domain z-scores. Finally, the centre point of the *best of the best* time-use zone was defined as the *Goldilocks day* composition. Refer to Fig 1 for a hypothetical example of how the five *best* time-use zones, five *optimal* time-use compositions, *best of the best* time-use zone, and *Goldilocks day* time-use composition are related. LPA and MVPA were merged into PA in the figure so that it could be presented as a 2-dimensional ternary plot.

**Fig 1:**
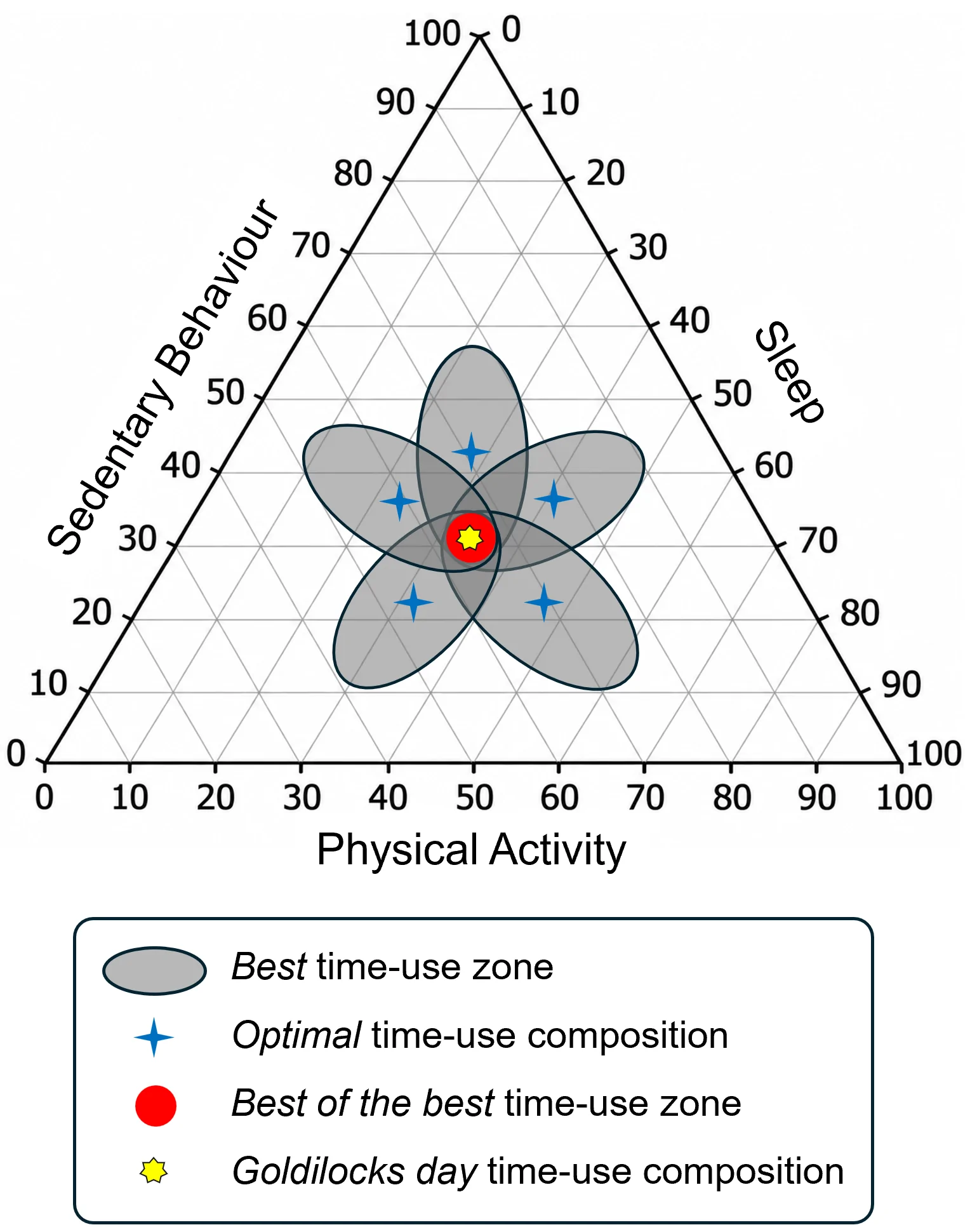
Hypothetical example of how the five *best* time-use zones, five *optimal* time-use compositions, *best of the best* time-use zone, and *Goldilocks day* time-use compositions relate to each other. Each axis in the ternary plot represents the percentage of the 24 hour day spent in each behaviour.

## Results

### Descriptive characteristics

After removing children with missing parent-reported sleep duration data, insufficient accelerometer data for assessment of PA and SED, and/or missing data for the social-emotional development outcomes, 2,060 children were examined: 560 children aged 1.2 to 2.9 years and 1,500 children aged 3.0 to 4.9 years (Fig 2). Key sociodemographic characteristics were similar for those included and excluded from the analyses (S3 Table in S1 File).

**Fig 2:**
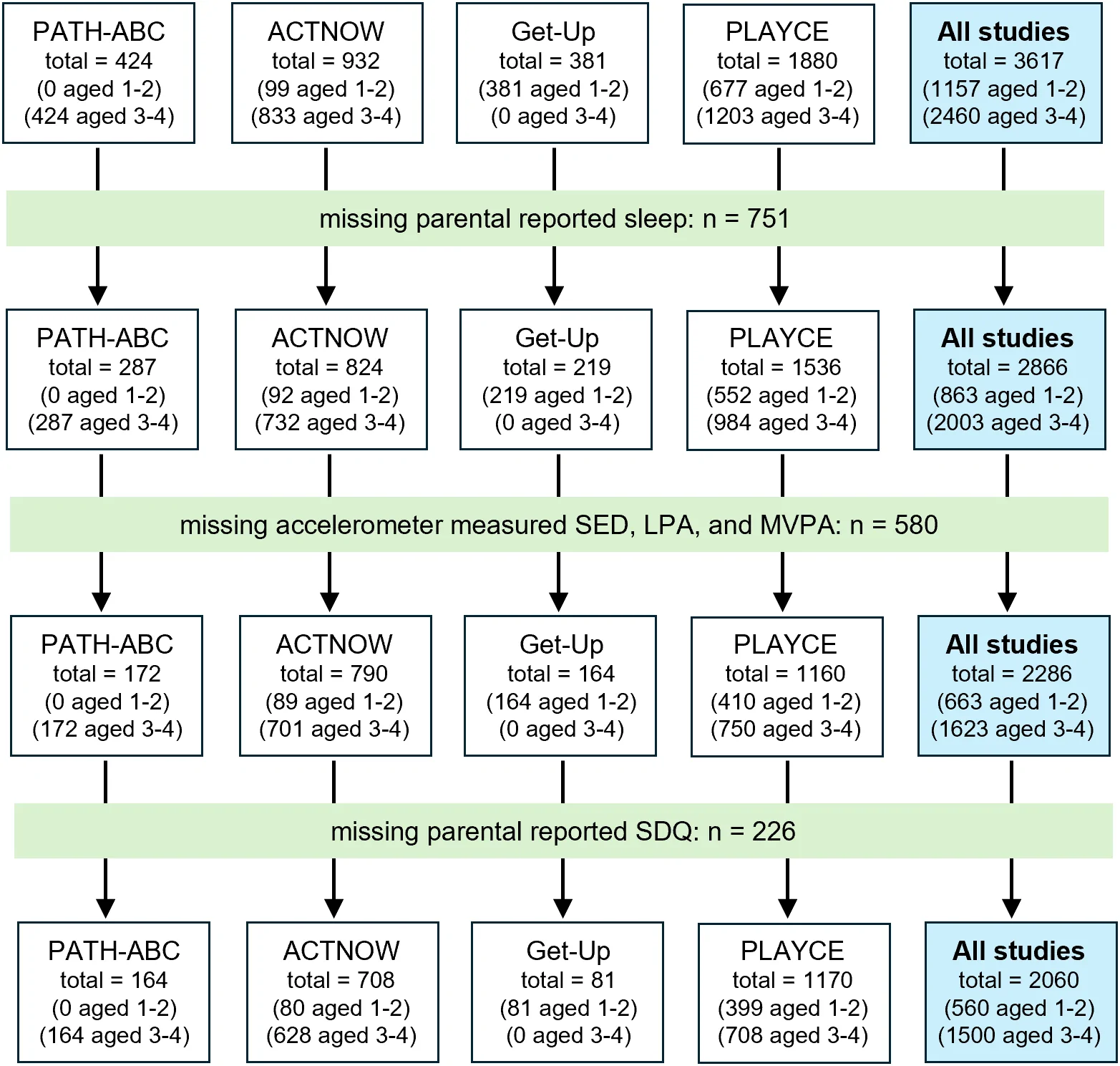
Flow chart illustrating exclusion of participants with missing data. SED, sedentary behaviour; LPA, light physical activity; MVPA, moderate-to-vigorous physical activity; SDQ, Strengths and Difficulties Questionnaire.

The descriptive characteristics of the analytical sample are provided in Table 1. Slightly more than half were boys, and most were from dual-parent households with well-educated parents. One to 2-year-olds accumulated 10 h 51 min of sleep, 5 h 46 min of SED, 5 h 55 min of LPA, and 1 h 28 min of MVPA per day on average. Three to 4-year-olds accumulated 10 h 40 min of sleep, 5 h 35 min of SED, 6 h 06 min of LPA, and 1h 39 min of MVPA per day on average. Median raw SDQ scores were 4 out of 10 or lower for the emotional problems, conduct problems, hyperactivity, and peer problems domains, and 7 out of 10 or higher for the prosocial behaviour domain.

**Table 1:** Participant characteristics.

Variable	1–2-year-olds, n (%) or mean (SD)	3–4-year-olds, n (%) or mean (SD)
Sex, n (%)		
Boys	303 (54.1)	785 (52.3)
Girls	257 (45.9)	715 (47.7)
Age, mean (SD)	2.4 (0.5)	3.90 (0.5)
Parental education, n (%)		
< high school	11 (2.5)	30 (2.0)
≥ high school	549 (97.5)	1470 (98.0)
Marital status, n (%)		
Dual parent	516 (92.1)	1353 (90.2)
Single parent	44 (7.9)	147 (9.8)
Social-emotional development scores, median (IQR)		
Emotional symptoms	1 (0-2)	1 (0-2)
Conduct problems	2 (1-3)	1 (0-3)
Hyperactivity	4 (2-5)	3 (2-5)
Peer problems	1 (0-2)	1 (0-2)
Prosocial behaviour	7 (6-8)	8 (6-9)
Movement behaviours*		
Sleep	10 h 51 min	10 h 40 min
Sedentary time	5 h 46 min	5 h 35 min
Light physical activity	5 h 55 min	6 h 06 min
Moderate-to-vigorous physical activity	1 h 28 min	1 h 39 min

SD: standard deviation; IQR: interquartile range.*Presented as compositional centre. Calculated as the geometric means of each activity adjusted so that the total sum was exactly 24 hours.

The percent agreement between top and non-top scores for the different SDQ domain z-scores are presented in S4 Table in S1 File. There was moderate to excellent agreement between top scores for emotional problems, conduct problems, hyperactivity, and peer problems while low agreement between the top scores for prosocial behaviour and the other four SDQ domain z-scores. Thus, the participants used to identify the *best* time-use zones were mostly the same for the emotional problems, conduct problems, hyperactivity, and peer problems domains but mostly different for the prosocial behaviour domain.

### Associations between movement behaviours and SDQ z-scores

Quadratic terms improved model fit and therefore were retained, indicating non-linear associations between movement behaviour compositions and the five SDQ domains. Estimates of these associations are presented in Table 2. The coefficients from these regression models represent the association for time spent in each movement behaviour relative to time spent in the remaining movement behaviours. Since higher SDQ z-scores indicate poorer social-emotional development for emotional symptoms, conduct problems, hyperactivity, and peer problems, negative coefficients reflect beneficial associations while positive coefficients reflect detrimental associations. Conversely, for prosocial behaviour, where higher scores are favorable, positive coefficients reflect desirable associations while negative coefficients reflect detrimental associations.

**Table 2:** Associations between movement behaviour compositions and sex- and age-standardized SDQ z-scores.

Age group and SDQ domain	Model P- value	Adjusted R² for ilrs	Sleep, β (95% CI)	SED, β (95% CI)	LPA, β (95% CI)	MVPA, β (95% CI)
**1–2-year-olds**						
Emotional symptoms	<0.001	0.150	−1.32 (−1.77, −0.87) *	8.41 (6.25, 10.57)*	−3.16 (−4.88, −1.44) *	0.80 (0.51, 1.09)*
Conduct problems	<0.001	0.129	−1.65 (−2.10, −1.20) *	1.50 (1.07, 1.93)*	−0.53 (−1.14, 0.08)	0.68 (0.37, 0.99)*
Hyperactivity	<0.001	0.090	−1.37 (−1.84, −0.90) *	1.06 (0.63, 1.49)*	−0.23 (−0.84, 0.38)	0.55 (0.24, 0.86)*
Peer problems	<0.001	0.129	−1.33 (−1.78, −0.88) *	1.53 (1.10, 1.96)*	−0.82 (−1.43, −0.21) *	0.62 (0.31, 0.93)*
Prosocial behaviour	<0.001	0.104	−1.28 (−1.73, −0.83) *	1.24 (1.00, 1.48)*	−0.53 (−1.12, 0.06)	0.57 (0.28, 0.86)*
**3–4-year-olds**						
Emotional symptoms	<0.001	0.139	−9.65 (−11.53, −7.77) *	16.00 (13.59, 18.41)*	−0.07 (−1.97, 1.83)	1.05 (−1.13, 3.23)
Conduct problems	<0.001	0.130	−11.01 (−12.87, −9.15) *	15.70 (13.35, 18.05)*	−0.29 (−2.15, 1.57)	2.39 (0.21, 4.57)*
Hyperactivity	<0.001	0.090	−9.20 (−11.10, −7.30) *	12.19 (9.80, 14.58)*	−0.11 (−1.99, 1.77)	3.02 (0.82, 5.22)*
Peer problems	<0.001	0.151	−11.11 (−12.95, −9.27) *	17.10 (14.79, 19.41)*	−0.29 (−2.11, 1.53)	1.54 (−0.60, 3.68)
Prosocial behaviour	<0.001	0.093	−7.04 (−9.00, −5.08) *	13.55 (11.08, 16.02)*	−2.25 (−4.21, −0.29) *	1.16 (−1.11, 3.43)

SDQ: Strengths and Difficulties Questionnaire; ilr: isometric log-ratio; SED: sedentary behaviour; LPA: light physical activity; MVPA: moderate-to-vigorous physical activity; CI: confidence interval. All β estimates are ilr regression coefficients that capture the relationships of one behaviour relative to the remaining behaviours. All estimates are adjusted for parental education, parental marital status, and study.* P < 0.05.

In both age groups, the movement behaviour composition was significantly associated with all five SDQ domains (p ≤ 0.01). Among 1–2-year-olds, relative time spent in sleep was beneficially associated with four SDQ z-scores, relative time in SED and MVPA were detrimentally associated with four SDQ z-scores, and relative time in LPA was beneficially associated with two SDQ z-scores. Among 3–4-year-olds, relative time spent in sleep was beneficially associated with four SDQ z-score, relative time in SED was detrimentally associated with 4 SDQ z-scores, relative time spent in LPA was detrimentally associated with prosocial behaviour, and relative time spent in MVPA was detrimentally with conduct problems and hyperactivity.

### Best time-use zones and optimal time-use movement behaviour compositions for SDQ domain z-scores

The *best* time-use zones (i.e., range of time-use compositions associated with the top scores for each domain) and *optimal* time-use compositions (i.e., compositional mean of the *best* time-use zones) are presented in Table 3. The percentage of children falling within the *best* time-use zones is presented in S5 Table in S1 File. Within each age group, the *best* time-use zones and *optimal* time-use compositions were comparable for the emotional symptoms, conduct problems, hyperactivity, and peer problems domains. The *best* time-use zones and *optimal* time-use compositions were noticeably different for the prosocial behaviour domain with higher values for SED and lower values for sleep. For example, for 3–4-year-olds, the *optimal* time use composition for prosocial behaviour consisted of 9 h 47 min of sleep, 7 h 0 min of SED, 5 h 55 min of LPA, and 1 h 18 min of MVPA. The corresponding values for the other four SDQ domains range from 12 h 0 min to 12 h 06 min for sleep, 4 h 24 min to 4 h 36 min for SED, 5 h 48 min to 6 h 0 min for LPA, and 1 h 30 min to 1 h 42 min for MVPA.

**Table 3:** Best time-use zones and optimal time-use compositions for the five sex- and age-standardized SDQ z-scores.

Age group and SDQ domain	Sleep		SED		LPA		MVPA	
	*Best* time-use zone	*Optimal* time-use composition	*Best* time-use zone	*Optimal* time-use composition	*Best* time-use zone	*Optimal* time-use composition	*Best* time-use zone	*Optimal* time-use composition
**1–2-year-olds**								
Emotional symptoms	10 h 48 min – 13 h 12 min	12 h 06 min	4 h 0 min – 5 h 36 min	4 h 36 min	4 h 48 min – 7 h 06 min	5 h 54 min	0 h 48 min – 2 h 12 min	1 h 24 min
Conduct problems	11 h 12 min – 13 h 12 min	12 h 18 min	4 h 0 min – 5 h 54 min	4 h 36 min	4 h 42 min – 6 h 54 min	5 h 54 min	0 h 48 min – 2 h 12 min	1 h 12 min
Hyperactivity	10 h 54 min – 13 h 12 min	12 h 12 min	4 h 0 min – 6 h 0 min	4 h 42 min	4 h 42 min – 7 h 0 min	5 h 48 min	0 h 48 min – 2 h 12 min	1 h 18 min
Peer problems	10 h 30 min – 13 h 12 min	12 h 06 min	4 h 0 min – 5 h 42 min	4 h 36 min	4 h 42 min – 7 h 12 min	5 h 54 min	0 h 48 min – 2 h 12 min	1 h 24 min
Prosocial behaviour	8 h 42 min – 11 h 48 min	9 h 42 min	4h 48 min – 7 h 12 min	5 h 54 min	4 h 48 min – 7 h 30 min	6 h 24 min	1 h 0 min – 2 h 48 min	2 h 0 min
**3–4-year-olds**								
Emotional symptoms	10 h 48 min – 12 h 48 min	12 h 0 min	3 h 54 min – 5 h 12 min	4 h 24 min	4 h 54 min – 7 h 12 min	6 h 0 min	1 h 12 min – 2 h 30 min	1 h 36 min
Conduct problems	11 h 0 min – 12 h 48 min	12 h 06 min	3 h 54 min – 5 h 18 min	4 h 24 min	4 h 48 min – 7 h 0 min	5 h 48 min	1 h 06 min – 2 h 24 min	1 h 42 min
Hyperactivity	10 h 54 min – 12 h 48 min	12 h 06 min	3 h 54 min – 5 h 30 min	4 h 36 min	4 h 48 min – 7 h 0 min	5 h 48 min	1 h 0 min – 2 h 12 min	1 h 30 min
Peer problems	11 h 06 min – 12 h 48 min	12 h 06 min	3 h 54 min – 5 h 18 min	4 h 24 min	4 h 48 min – 6 h 54 min	5 h 48 min	1 h 06 min – 2 h 30 min	1 h 42 min
Prosocial behaviour	8 h 42 min – 11 h 24 min	9 h 47 min	6 h 24 min – 7 h 42 min	7 h 0 min	4 h 48 min – 7 h 12 min	5 h 55 min	0 h 54 min – 1 h 48 min	1 h 18 min

SDQ: Strengths and Difficulties Questionnaire; SED: sedentary behaviour; LPA: light physical activity; MVPA: moderate-to-vigorous physical activity.

### Best of the best time-use zone and Goldilocks day time-use composition for SDQ domain z-scores

The *best of the best* time-use zone (i.e., overlapping area of the five *best* time-use zones) and *Goldilocks day* time-use composition (i.e., compositional mean of the *best of the best* time-use zone) are presented in Table 4. The percentage of children falling within the *best of the best* time-use zones is presented in S5 Table in S1 File. In 1–2-year-olds, the *Goldilocks day* time-use composition consisted of 12 h 12 min of sleep, 4 h 36 min of SED, 5 h 48 min of LPA, and 1h 24 min of MVPA (Fig 3). The corresponding values for 3–4-year-olds were 12 h 08 min, 4 h 27 min, 5 h 49 min, and 1 h 36 min, respectively (Fig 3).

**Table 4:** Best of the best time-use zones and Goldilocks day time-use composition for social-emotional functioning.

Age group	Sleep		SED		LPA		MVPA	
	*Best of the best* time-use zone	*Goldilocks day* time-use composition	*Best of the best* time-use zone	*Goldilocks day* time-use composition	*Best of the best* time-use zone	*Goldilocks day* time-use composition	*Best of the best* time-use zone	*Goldilocks day* time-use composition
1–2-year-olds	10 h 42 min – 13 h 12 min	12 h 12 min	4 h 0 min – 5 h 54 min	4 h 36 min	4 h 42 min – 7 h 06 min	5 h 48 min	0 h 48 min – 2 h 12 min	1 h 24 min
3–4-year-olds	11 h 00 min – 12 h 50 min	12 h 08 min	3 h 54 min – 5 h 18 min	4 h 27 min	4 h 48 min – 7 h 0 min	5 h 49 min	1 h 05 min – 2 h 30 min	1 h 36 min

SED: sedentary behaviour; LPA: light physical activity; MVPA: moderate-to-vigorous physical activity.

**Fig 3:**
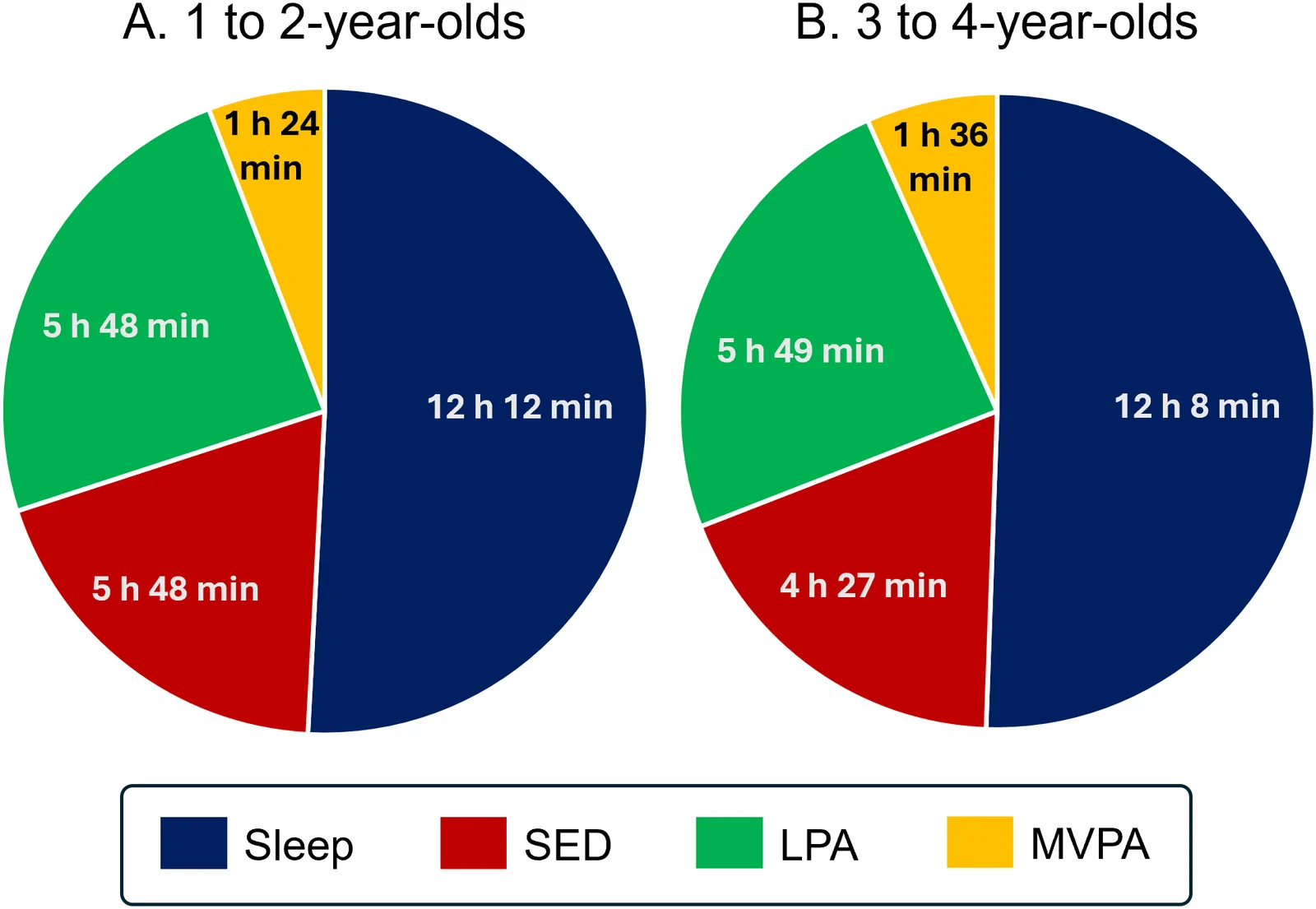
Goldilocks day-time-use composition for desired social-emotional functioning in the early years. SED, sedentary time; LPA, light physical activity; MVPA, moderate-to-vigorous physical activity.

### Associations between movement behaviours and SDQ z-scores determined using traditional regression

Some of the CoDA regression findings presented in Table 2 were unexpected and inconsistent with prior research that used traditional regression approaches. Specifically, there were detrimental associations between MVPA and four SDQ z-scores among 1–2-year-olds and two SDQ z-scores 3–4-year-olds. Therefore, we conducted an a posteriori analysis using a traditional, non-CoDA approach where the movement behaviours were treated as independent variables in a multivariable linear regression model that included time spent in each movement behaviour and the confounding variables. S6 Table in S1 File shows the associations for each movement behaviour after adjusting for confounding variables but not the remaining movement behaviours (Model 1), as well as after adjusting for confounding variables and each of the remaining movement behaviours (Model 2).

After adjusting for confounding variables only (Model 1), MVPA remained detrimentally associated with four SDQ z-scores among 1–2-year-olds but became insignificant for the two SDQ z-scores among 3–4-year-olds. Some of these associations changed after further adjusting for the remaining movement behaviours (Model 2). Specifically, among 3–4-year-olds, MVPA became beneficially associated with conduct problem.

## Discussion

### Principal findings and contributions

This study extends previous research by identifying 24-hour movement behaviour compositions associated with favourable social-emotional functioning in early years children. Rather than examining sleep, sedentary time, and physical activity as independent behaviours, the Goldilocks Day approach identifies combinations of these behaviours that simultaneously correspond with favourable scores across multiple social-emotional domains. The findings also provide age-specific estimates for 1- to 2-year-olds and 3- to 4-year-olds, thereby extending the limited CoDA literature examining movement behaviours and social-emotional development in the early years. Importantly, the identified best time-use zones were relatively broad, suggesting that favourable social-emotional functioning may be compatible with a range of daily movement behaviour compositions rather than a single optimal 24-hour schedule. Our CoDA analysis (see Table 2) revealed detrimental associations between SED and social-emotional functioning among 1–4-year-olds, which contradicts the results of our own analyses that used traditional regression approaches (see S6 Table in S1 File). This discrepancy may reflect that CoDA accounts for the compositional nature of movement behaviours by recognizing that the time spent in different movement behaviours is interdependent and constrained within a 24-hour period [40]. In the CoDA analyses, sleep had a particularly strong beneficial association with social-emotional development. A systematic review showed that shorter sleep duration in early childhood is independently associated with poorer emotional regulation [41] as sleep plays a crucial role in fostering essential social and emotional development.

### Comparison with previous CoDA studies

Results from previous studies that used CoDA to examine associations between 24-hour movement behaviours and social-emotional functioning indicators in the early years are inconsistent [8,10]. Arts et al. [42] found no associations between the overall composition (or individual movement behaviour components) and social-emotional functioning in cross-sectional (n = 62) and longitudinal (n = 101) analyses of children aged 0–4 years. However, interpretation of these null findings is limited by several methodological limitations including the subjective measures of movement behaviours, lack of differentiating physical activity by intensity (i.e., LPA versus MVPA), and a small sample size. Similarly, St. Laurent et al. (13) conducted cross-sectional analyses of 327–371 children aged 2.8–5.8 years and reported null associations between the overall movement behaviour composition and social-emotional outcomes. However, it is important to note that St. Laurent et al. study sample consisted of very healthy preschool children with very low behavioural problem scores (e.g., mean externalizing: 8.51 out of 44; mean internalizing: 5.11 out of 52). This restricted variance in social-emotional outcomes can limit statistical power to detect associations between movement behaviors and social-emotional health measures. Children in the present study also demonstrated generally favourable social-emotional functioning, with relatively low difficulties scores and high prosocial behaviour scores compared with published age-specific SDQ reference values [32,37,43]. Thus, the relatively healthy distribution of SDQ scores in our sample may also have reduced variability in the outcomes and limited our ability to detect some associations with movement behaviours.

In contrast, Phillips et al. [9] in a study of 107 preschool children (mean age: 3.4 ± 0.5 years), reported that the overall composition during childcare hours was not associated with social-emotional outcomes, although relative time in LPA was unfavourably associated with internalising problems and relative time in MVPA was favourably associated with internalising problems. However, no significant associations were observed between relative time spent in SED, LPA, and MVPA and other SDQ outcomes, including externalizing problems, prosocial behaviour, and total difficulties [9]. Similarly, Kuzik et al. [10] observed a favourable association between MVPA and sociability in preschool children (n = 95; mean age ≈ 4.5 years), with null associations for sleep, SED, and LPA. Li et al. [8] provided additional insight by differentiating screen-based and non-screen SED in a sample of 205 children aged 3–6 years. They reported that the overall movement behaviour composition was associated with prosocial behaviour and externalizing problems; relative time spent in sleep was unfavourably associated with prosocial behaviour, whereas relative time in non-screen SED was favourably associated with both prosocial behaviour and externalizing problems. No associations were observed for LPA or MVPA.

Overall, our findings are most consistent with those of Li et al. [8] both in terms of composition-level associations with social-emotional outcomes and in suggesting that the type of SED may be important. In our study, SED was generally unfavourably associated with most SDQ domains. Although we did not differentiate SED by type, it is possible that this association is driven in part by screen-based SED, as suggested in prior work [44]. In the present study, MVPA was associated with fewer social-emotional domains in the older age group compared with younger children, and associations were in the unexpected direction. This observation somewhat aligns with previous studies reporting null MVPA associations for several social-emotional outcomes and may reflect developmental differences or shifts in how MVPA displaces other behaviours within the constrained 24-hour day [8,9].

The variability in results across CoDA movement behaviour and socio-emotional functioning indicators in early years children likely reflects differences in developmental stage of the samples, socio-emotional indicators examined, measurement methods of both movement behaviours and socio-emotional functioning indicators, and in some case, small sample sizes that could have led to underpowered analyses. Collectively, this body of evidence challenges a simplistic binary interpretation that more movement is always beneficial, and more sitting is always detrimental. Instead, it highlights the importance of examining the relative allocation of time across the full 24-hour day to better understand how combinations of movement behaviours relate to social-emotional functioning in early childhood.

### Interpretation of the Goldilocks day

To our knowledge, only a single study of early years children has used the CoDA Goldilocks day analysis approach to investigate the relationships between movement behaviours and measures of cognition and social-emotional health [13]. In that study of 388 children aged 2.7–5.8 years, the movement behaviour composition was associated only with the receptive vocabulary measure. The *optimal* time-use for vocabulary scores was 12 h 06 min of sleep, 4 h 42 min of SED, 5 h 36 min of LPA, and 1 h 42 min of MVPA [13]. These values were comparable to the *optimal* time-use composition for desirable emotional symptoms, conduct problems, hyperactivity, and peer problems in our study. The similar *optimal* time-use values we observed for these four SDQ domains may reflect the good agreement between desirable scores for these four SDQ domains (see S4 Table in S1 File). Conversely, the *optimal* time-use for desirable prosocial behaviour scores, which had low agreement with the desirable scores for the other four SDQ domains, was noticeably different with higher SED and lower LPA and MVPA values. This may reflect that prosocial behaviour is a positive behaviour, whereas the other domains reflect negative developmental outcomes. Furthermore, given the multidimensional structure of the SDQ, time-use compositions associated with lower social-emotional difficulties may differ from those associated with higher prosocial behaviour. Accordingly, “optimal” in this study refers to domain-specific desirable scores rather than a single composition that simultaneously maximizes all aspects of social-emotional development.

In our study, the *Goldilocks day* values for sleep duration, which were 12 h 12 min for 1–2-year-olds and 12 h 08 min for 3–4-year-olds, were within the sleep duration ranges recommended in 24-hour Movement Guidelines, which are 11–14 hours for 1–2-year-olds and 10–13 hours for 3–4-year-olds [6,7,11,45,46]. However, the *Goldilocks day* values for total PA (LPA + MVPA), which were 7 h 12 min for 1–2-year-olds and 7 h 25 min for 3–4-year-olds, were considerably higher than the minimum PA target in the 24-hour Movement Guidelines, which is three hours for 1–4-year-olds [6,7,11,45,46].This difference likely reflects the distinct purposes of these approaches: movement guidelines define minimum thresholds intended to achieve broad population-level health benefits, whereas Goldilocks day analyses identify relative time-use patterns associated with more favourable outcomes within a specific dataset and for a specific developmental domain. Importantly, Goldilocks day estimates should not be interpreted as behavioural recommendations or targets, but rather as an analytical framework for exploring how combinations of movement behaviours across the 24-hour day relate to desired health outcomes.

The Goldilocks day sleep durations were also higher than the geometric mean sleep durations observed in the study sample (Table 1). This difference is expected because the geometric means describe the average movement behaviour profile of the entire study sample, whereas the Goldilocks day represents the statistical centre (compositional mean) of the movement behaviour compositions associated with the most favourable SDQ scores. Consequently, the Goldilocks day is meant to identify movement behaviour patterns associated with more favourable outcomes rather than to describe the average movement behaviour profile of the study population.

### Strengths and limitations

Our study provides a comprehensive insight into the optimal time-use compositions for all SDQ domains across two early years age groups. Our large sample size, the use of validated measures like the SDQ, as well as the use of device-based measurements further strengthen our findings. However, our study is limited by its cross-sectional design and inability to establish causal relationships. Some potential confounding factors, such as race and ethnicity, were unavailable for inclusion in our analysis. Furthermore, our study did not differentiate between screen-based and non-screen-based sedentary behaviour. In addition, non-wear time was treated as missing data and imputed proportionally across waking movement behaviours based on observed wear-time distributions; however, this imputation may not fully reflect true behaviour during non-wear periods. Finally, while the *Goldilocks day* approach offers valuable insights, it might not adequately represent the reality of children’s lives and could create unrealistic expectations for some children, teachers, and caregivers.

## Conclusion

This study presents movement behaviour values and zones associated with the best social-emotional functioning in early years children. The findings highlight that optimal movement behaviour levels may be quite different than current guideline recommendations. With that said, the Goldilocks day estimates should be interpreted as hypothesis-generating statistical summaries rather than prescriptive behavioural or public health recommendations. Further research using longitudinal and experimental designs is needed to examine how different combinations of sleep, SED, LPA, and MVPA relate to social-emotional functioning and other health outcomes across early childhood. Further research might also consider expanding on the Goldilocks day approach to provide information about different combinations of these movement behaviors that could result in suboptimal scores (i.e., scores that are beneficial but not optimal).

## Supporting information

S1 File Supplemental file with tables that provide additional methods and results not presented in the manuscript. (DOCX)

## Abbreviations

PA: Physical activity
SED: Sedentary behaviour
LPA: Light physical activity
MVPA: Moderate-to-vigorous physical activity
SDQ: Strengths and Difficulties Questionnaire
CoDA: Compositional data analysis
SADEY: Sleep and Activity Database for the Early Years
ilr: isometric log-ratio
