# Supplementary material for: 24-hour movement behaviour composition associated with desired social-emotional functioning in the early years
Source: PLoS One. 2026 Oct 21;21(10):e0361046. doi: 10.1371/journal.pone.0361046 (PMC13686454; doi:10.1371/journal.pone.0361046)
Supplement: Supplementary file 1 [file pone.0361046.s001.docx]

**Supplementary File**

Table S1 ……………………………………………………………………………………. page 2

Table S2 ……………………………………………………………………………………. page 3

Table S3 ……………………………………………………………………………………. page 4

Table S4 ……………………………………………………………………………………. page 5

Table S5 ……………………………………………………………………………………. page 6

Table S6 ……………………………………………………………………………………. page 7

| **Table S1:** Strengths and Difficulties Questionnaire (SDQ) domains, individual items, and scoring system used to derive domain summary scores. | | | |
| --- | --- | --- | --- |
|  | **Response Option and Score** | | |
| **SDQ domains and items** | **Not**  **True** | **Somewhat True** | **Certainly True** |
| Emotional symptoms |  |  |  |
| Often complains of headaches, stomach-ache or sickness | 0 | 1 | 2 |
| Many worries, often seems worried. | 0 | 1 | 2 |
| Often unhappy, down-hearted or tearful | 0 | 1 | 2 |
| Nervous or clingy in new situation, easily loses confidence | 0 | 1 | 2 |
| Many fears easily scared. | 0 | 1 | 2 |
|  |  |  |  |
| Conduct problems |  |  |  |
| Often has temper tantrums or hot tempers | 0 | 1 | 2 |
| Generally obedient, usually does what adults request | 2 | 1 | 0 |
| Often fights with other children or bullies them | 0 | 1 | 2 |
| Often lies or cheats | 0 | 1 | 2 |
| Steals from home, school, or elsewhere | 0 | 1 | 2 |
|  |  |  |  |
| Hyperactivity |  |  |  |
| Restless, overactive, cannot stay still for long | 0 | 1 | 2 |
| Constantly fidgeting or squirming | 0 | 1 | 2 |
| Easily distracted, concentration wanders | 0 | 1 | 2 |
| Thinks things out before acting | 2 | 1 | 0 |
| Sees tasks through to the end, good attention span | 2 | 1 | 0 |
|  |  |  |  |
| Peer problems |  |  |  |
| Rather solitary, tends to play alone | 0 | 1 | 2 |
| Has at least one good friend | 2 | 1 | 0 |
| Generally liked by other children | 2 | 1 | 0 |
| Picked on or bullied by other children | 0 | 1 | 2 |
| Gets on better with adults than with other children | 0 | 1 | 2 |
|  |  |  |  |
| Prosocial Behaviour |  |  |  |
| Considerate of other people's feelings | 0 | 1 | 2 |
| Shares readily with other children (treats, toys, pencils, etc.) | 0 | 1 | 2 |
| Helpful if someone is hurt, upset, or feeling ill | 0 | 1 | 2 |
| Kind to younger children | 0 | 1 | 2 |
| Volunteers to help others (parents, teachers, other children) | 0 | 1 | 2 |
| Note: Scores are added in each domain to create a summary | | | |

| **Table S2:** Harmonization of parental education and marital status variables across contributing studies included in the SADEY consortium. | | |
| --- | --- | --- |
| **Study** | **Categories used in original studies** | **Harmonized categories used in SADEY** |
| **Parental education** | | |
| PATH-ABC | 1 = No formal education  2 = Year 10 or equivalent  3 = Year 12 or equivalent  4 = Trade/Apprenticeship/Certificate  5 = Diploma  6 = University degree  7 = Post-graduate qualification | 1 = ≤ high school  1 = ≤ high school  1 = ≤ high school  2 = > high school  2 = > high school  2 = > high school  2 = > high school |
| ACTNOW | 1 = Primary school (9 years)  2 = High school (12-13 years)  3 = University | 1 = ≤ high school  1 = ≤ high school  2 = > high school |
| Get-Up | 1 = No schooling/did not complete Primary School  2 = Primary school or equivalent  3 = Year 10 or equivalent  4 = Year 12 or equivalent  5 = Trade/apprenticeship/certificate  6 = University Degree  7 = Post-graduate qualification | 1 = ≤ high school  1 = ≤ high school  1 = ≤ high school  1 = ≤ high school  2 = > high school  2 = > high school  2 = > high school |
| PLAYCE | 1 = Year 12 or lower  2 = Trade/diploma  3 = University or Postgrad | 1 = ≤ high school  2 = > high school  2 = > high school |
| **Marital status** | | |
| PATH-ABC | 1 = Never married, single parent  2 = Never married, Live with partner  3 = Married, live with partner  4 = Separated/divorced  5 = Widowed | 1 = single parent  2 = dual parent  2 = dual parent  1 = single parent  1 = single parent |
| ACTNOW | 1 = Married/cohabitant  2 = Alone/divorced/widow | 2 = dual parent  1 = single parent |
| Get-Up | 1 = Single/separated/divorced  2 = Married/with partner | 1 = single parent  2 = dual parent |
| PLAYCE | 1 = Married  2 = Defacto  3 = Separated/Divorced  4 = Widowed  5 = Single, never married | 2 = dual parent  2 = dual parents  1 = single parent  1 = single parent  1 = single parent |

| **Table S3:** Socio-demographic characteristics of SADEY participants included and excluded from the analyses, stratified by age group. | | | | |
| --- | --- | --- | --- | --- |
| **Variable** | **1 to 2-year-olds** | | **3 to 4-year-olds** | |
|  | **Included** | **Excluded** | **Included** | **Excluded** |
| Age, mean (SD) | 2.3 (0.5) | 1.8 (0.5) * | 3.8 (0.6) | 3.8 (0.6) |
| Sex, % | | | | |
| Boys | 54.3 | 56.3 | 51.3 | 54.4 |
| Girls | 45.7 | 43.7 | 48.7 | 45.6 |
| Parental education, % | | | | |
| ≤ high school | 0.6 | 0.3 | 2.0 | 3.2 |
| > high school | 99.4 | 99.7 | 98.0 | 96.8 |
| Marital status, % | | | | |
| Dual parent | 92.1 | 88.8 | 89.9 | 89.5 |
| Single parent | 7.9 | 11.2 | 10.1 | 10.5 |
| * p<0.05 versus Included. | | | | |

| **Table S4:** Percentage agreement between top and non-top scores across Strengths and Difficulties Questionnaire (SDQ) domain z-scores for children aged 1–2 and 3–4 years. | | | | |
| --- | --- | --- | --- | --- |
|  | **Conduct problems** | **Hyperactivity** | **Peer problems** | **Prosocial behaviour** |
| **1 to 2-year-olds** |  |  |  |  |
| Emotional problems | 98.6% | 97.6% | 99.3% | 31.7% |
| Conduct problems | - | 92.7% | 89.5% | 9.3% |
| Hyperactivity | - | - | 53.1% | 11% |
| Peer problems | - | - | - | 9.1% |
| Prosocial behaviour | - | - | - | - |
| **3 to 4-year-olds** | | | | |
| Emotional problems | 71.8% | 70.4% | 71.6.9% | 13.9% |
| Conduct problems | - | 85.4% | 73.1% | 26.4% |
| Hyperactivity | - | - | 63.3% | 29.2% |
| Peer problems | - | - | - | 21.5% |
| Prosocial behaviour | - | - | - | - |

| **Table S5:** Percentage of participants falling within the *best* time-use zone for the individual Strengths and Difficulties Questionnaire (SDQ) domains and the overall *best of the best* time-use zone. | | | | |
| --- | --- | --- | --- | --- |
| **Age group and SDQ domain** | **Sleep** | **Sedentary behaviour** | **Light physical activity** | **Moderate-to-vigorous physical activity** |
| **1 to 2-year-olds** |  |  |  |  |
| Emotional symptoms | 58.7% | 59.3% | 92.3% | 89.3% |
| Conduct problems | 32.3% | 59.3% | 92.2% | 87.1% |
| Hyperactivity | 57.3% | 63.3% | 92.5% | 87.1% |
| Peer problems | 71.6% | 45.8% | 93.5% | 89.3% |
| Prosocial behaviour | 76.6% | 78.2% | 94.1% | 87.6% |
| *Best of the best* time-use zone | 58.7% | 59.3% | 92.8% | 89.3% |
| **3 to 4-year-olds** | | | | |
| Emotional symptoms | 55.4% | 33.7% | 91.5% | 87.3% |
| Conduct problems | 54.9% | 40.9% | 90% | 85.7% |
| Hyperactivity | 54.9% | 47.3% | 90% | 80.4% |
| Peer problems | 23.3% | 40.9% | 88.3% | 87.2 % |
| Prosocial behaviour | 73.6 % | 14.6 % | 92.7 % | 62.6 % |
| *Best of the best* time-use zones | 54.9% | 40.9% | 90% | 87.2 % |

| **Table S6.** Linear regression model estimates of the independent associations between movement behaviours and Strengths and Difficulties Questionnaire (SDQ) domain z-scores, stratified by age group. | | | | | |
| --- | --- | --- | --- | --- | --- |
| **Age group and SDQ domain** | **Model** | **Sleep, β (95% CI)** | **SED, β (95% CI)** | **LPA, β (95% CI)** | **MVPA, β (95% CI)** |
| **1 to 2-year-olds** |  |  |  |  |  |
| Emotional symptoms | Model 1 | -0.003* (-0.004, -0.002) | -0.003* (-0.005, -0.001) | -0.000 (-0.002, 0.002) | 0.004* (0.002, 0.006) |
|  | Model 2 | -0.009* (-0.013, -0.005) | -0.003* (-0.005, -0.001) | -0.009* (-0.013, -0.005) | 0.003* (0.001, 0.005) |
| Conduct problems | Model 1 | -0.004* (-0.006, -0.002) | 0.003* (0.001, 0.005) | 0.001 (-0.001, 0.003) | 0.004* (0.001, 0.007) |
|  | Model 2 | -0.008* (-0.011, -0.005) | -0.002 (-0.005, 0.001) | -0.007* (-0.011, -0.004) | 0.002 (-0.001, 0.005) |
| Hyperactivity | Model 1 | -0.003* (-0.005, -0.001) | 0.002* (0.000, 0.004) | 0.001 (-0.001, 0.003) | 0.003* (0.001, 0.005) |
|  | Model 2 | -0.006* (-0.009, -0.003) | 0.002* (0.000, 0.004) | -0.005* (-0.008, -0.002) | 0.002 (-0.001, 0.005) |
| Peer problems | Model 1 | -0.003* (-0.005, -0.001) | 0.002* (0.000, 0.004) | 0.001 (-0.001, 0.003) | 0.003* (0.001, 0.005) |
|  | Model 2 | -0.006* (-0.009, -0.003) | -0.002 (-0.004, 0.001) | -0.005* (-0.008, -0.002) | 0.002 (-0.001, 0.005) |
| Prosocial behaviour | Model 1 | -0.003* (-0.005, -0.001) | 0.003* (0.001, 0.005) | 0.000 (-0.002, 0.002) | 0.003* (0.001, 0.005) |
|  | Model 2 | -0.007* (-0.010, -0.004) | -0.002 (-0.005, 0.001) | -0.006* (-0.009, -0.003) | 0.002 (-0.001, 0.005) |
| **3 to 4-year-olds** |  |  |  |  |  |
| Emotional symptoms | Model 1 | -0.004* (-0.005, -0.003) | 0.006* (0.004, 0.008) | -0.000 (-0.001, 0.001) | -0.003* (-0.005, -0.001) |
|  | Model 2 | -0.002* (-0.003, -0.001) | 0.004* (0.002, 0.006) | -0.000 (-0.002, 0.002) | -0.004* (-0.006, -0.002) |
| Conduct problems | Model 1 | -0.004* (-0.005, -0.003) | 0.005* (0.003, 0.007) | 0.000 (-0.001, 0.001) | -0.001 (-0.003, 0.001) |
|  | Model 2 | -0.004* (-0.006, -0.002) | 0.003* (0.001, 0.005) | -0.002 (-0.004, 0.000) | -0.003* (-0.005, -0.001) |
| Hyperactivity | Model 1 | -0.004* (-0.005, -0.003) | 0.004 (-0.000, 0.008) | 0.000 (-0.001, 0.001) | -0.000 (-0.002, 0.002) |
|  | Model 2 | -0.004* (-0.006, -0.002) | 0.001 (-0.001, 0.003) | -0.002* (-0.004, -0.000) | -0.001 (-0.003, 0.001) |
| Peer problems | Model 1 | -0.005* (-0.007, -0.003) | 0.006* (0.004, 0.008) | 0.000 (-0.001, 0.001) | -0.003* (-0.005, -0.001) |
|  | Model 2 | -0.007* (-0.009, -0.005) | 0.004* (0.002, 0.006) | -0.005* (-0.007, -0.003) | -0.004* (-0.006, -0.002) |
| Prosocial behaviour | Model 1 | -0.003* (-0.004, -0.002) | 0.005* (0.003, 0.007) | -0.000 (-0.001, 0.001) | -0.002* (-0.004, -0.000) |
|  | Model 2 | -0.002* (-0.003, -0.001) | 0.003* (0.001, 0.005) | -0.002 (-0.004, 0.000) | -0.003* (-0.005, -0.001) |
| SDQ: Strength and Difficulties Questionnaire; SED: sedentary behaviour; LPA: light physical activity; MVPA: moderate-to-vigorous physical activity; β: unstandardized regression coefficient.  Model 1: Adjusted for parental education, parental marital status, and study.  Model 2: Adjusted for the remaining movement behaviours, parental education, parental marital status, and study.  ***** P<0.05.  Variance inflation factors for model 1 for 1 to 2-year-olds: sleep 1.010, SED 1.020, LPA 1.025, MVPA 1.048.  Variance inflation factors for model 2 for 1 to 2-year-olds: sleep 1.604, SED 1.257, LPA 1.154, MVPA 1.396.  Variance inflation factors for model 1 for 3 to 4-year-olds: sleep 1.007, SED 1.017, LPA 1.018, MVPA 1.052.  Variance inflation factors for model 2 for 3 to 4-year-olds sleep 1.580, SED 1.320, LPA 1.097 MVPA: 1.399. | | | | | |
